# Effect of Cholesterol on Membrane Fluidity and Association of Aβ Oligomers and Subsequent Neuronal Damage: A Double-Edged Sword

**DOI:** 10.3389/fnagi.2018.00226

**Published:** 2018-08-03

**Authors:** Eduardo J. Fernández-Pérez, Fernando J. Sepúlveda, Christian Peters, Denisse Bascuñán, Nicolás O. Riffo-Lepe, Juliana González-Sanmiguel, Susana A. Sánchez, Robert W. Peoples, Benjamín Vicente, Luis G. Aguayo

**Affiliations:** ^1^Laboratory of Neurophysiology, Department of Physiology, Universidad de Concepción, Concepción, Chile; ^2^Departamento de Polímeros, Facultad de Ciencias Químicas, Universidad de Concepción, Concepción, Chile; ^3^Department of Biomedical Sciences, Marquette University, Milwaukee, WI, United States; ^4^Department of Psychiatry and Mental Health, Universidad de Concepción, Concepción, Chile

**Keywords:** Alzheimer’s disease, amyloid beta, membrane perforation, amyloid pore, membrane lipids, membrane fluidity, cholesterol

## Abstract

**Background:** The beta-amyloid peptide (Aβ) involved in Alzheimer’s disease (AD) has been described to associate/aggregate on the cell surface disrupting the membrane through pore formation and breakage. However, molecular determinants involved for this interaction (e.g., some physicochemical properties of the cell membrane) are largely unknown. Since cholesterol is an important molecule for membrane structure and fluidity, we examined the effect of varying cholesterol content with the association and membrane perforation by Aβ in cultured hippocampal neurons.

**Methods:** To decrease or increase the levels of cholesterol in the membrane we used methyl-β-cyclodextrin (MβCD) and MβCD/cholesterol, respectively. We analyzed if membrane fluidity was affected using generalized polarization (GP) imaging and the fluorescent dye di-4-ANEPPDHQ. Additionally membrane association and perforation was assessed using immunocytochemistry and electrophysiological techniques, respectively.

**Results:** The results showed that cholesterol removal decreased the macroscopic association of Aβ to neuronal membranes (fluorescent-puncta/20 μm: control = 18 ± 2 vs. MβCD = 10 ± 1, *p* < 0.05) and induced a facilitation of the membrane perforation by Aβ with respect to control cells (half-time for maximal charge transferred: control = 7.2 vs. MβCD = 4.4). Under this condition, we found an increase in membrane fluidity (46 ± 3.3% decrease in GP value, *p* < 0.001). On the contrary, increasing cholesterol levels incremented membrane rigidity (38 ± 2.7% increase in GP value, *p* < 0.001) and enhanced the association and clustering of Aβ (fluorescent-puncta/20 μm: control = 18 ± 2 vs. MβCD = 10 ± 1, *p* < 0.01), but inhibited membrane disruption.

**Conclusion:** Our results strongly support the significance of plasma membrane organization in the toxic effects of Aβ in hippocampal neurons, since fluidity can regulate distribution and insertion of the Aβ peptide in the neuronal membrane.

## Introduction

Alzheimer’s disease (AD) is a progressive and likely multifactorial disease that affects nearly 50 million people all over the world (Alzheimer’s-Disease-International, 2015). For decades, the deposition of extracellular amyloid-beta peptide (Aβ) has been a pathological hallmark of the disease and is believed to be one of the main pathological agents of the illness (Kumar et al., 2015; Sadigh-Eteghad et al., 2015). Aβ originates from the proteolytic processing of the APP, which has proteolytic sites for three enzymes, α-, β-, and γ- secretases, with the latter two being responsible for the generation of Aβ (Kumar et al., 2015; Bolduc et al., 2016). Although an increase in brain Aβ can explain part of the neurodegeneration detected in the AD brain, other toxic mediators, such as tau hyper phosphorylation (Swerdlow, 2007; Masters et al., 2015), oxidative (Kanamaru et al., 2015), and endoplasmic reticulum stress (Hashimoto and Saido, 2018), mitochondrial dysfunction and glutamatergic-induced excitotoxicity (Olloquequi et al., 2018), also likely contribute to disease progression.

Several risk factors for this disease have been described, including (but not limited to): age (Beydoun et al., 2008), low education level (Grünblatt et al., 2009), smoking (Cataldo et al., 2010), obesity (Kivipelto et al., 2005; Beydoun et al., 2008), and diabetes mellitus (Arvanitakis et al., 2004). A significant body of evidence that has been widely discussed is that cholesterol metabolism might be implicated in AD. For instance, presence of the ε4 allele of APOE seems to be a major risk factor for this disease, specifically in the late-onset form of AD (Roses, 1996; Tanzi and Bertram, 2005). APOE has also been shown to have a role in the burden of Aβ in the brain, promoting its degradation (Jiang et al., 2008) and clearance (Xiong et al., 2008). Nevertheless, the role of cholesterol in Aβ neurotoxicity has been controversial. For example, it was reported that cholesterol can increase the binding of Aβ to artificial lipid membranes (Ji et al., 2002; Matsuzaki, 2007) and that its level is inversely related to the toxic effects of Aβ oligomers (Cecchi et al., 2009). It is also well recognized that cholesterol induces changes in the physicochemical properties of membranes, such as changes in fluidity and density packing of lipids in neuronal membranes, which may affect the binding of Aβ to the cell membrane (Mirzabekov et al., 1996; Yu and Zheng, 2012). In addition, other studies found that increased levels of cholesterol, in human neuroblastoma cells, reduced the ability of Aβ to bind to the membranes as demonstrated by co-localization of Aβ with GM1 ganglioside, a marker for lipid rafts (Cecchi et al., 2009). These results agree with previous data showing that an increase in cholesterol levels exerted a protective effect against the toxicity induced by Aβ oligomers in neuroblastoma cells (Cecchi et al., 2008). In addition, it has been found that Aβ oligomers were associated with DRMDs in a cholesterol-dependent manner in neurons (Schneider et al., 2006), mouse models (Kawarabayashi et al., 2004), and AD brains (Kawarabayashi et al., 2004), and that the depletion of cholesterol reduced the aggregation of Aβ (Schneider et al., 2006).

Taken together, these data suggest that changes in cholesterol levels can modulate the composition and physicochemical properties of lipid rafts altering the binding of Aβ to the membrane. We reasoned that it was relevant to study how the physicochemical properties of the membrane that depends on cholesterol levels might be implicated in the initial steps in which Aβ disrupts the plasma membrane. Therefore, we characterized how a crucial membrane component, such as cholesterol, affects the cellular and physiological effects observed when Aβ associated with the neuronal membranes. Using biophysical approaches, we wanted to characterize the role of cholesterol in membrane fluidity and to examine how fluidity changes might contribute to association and formation of Aβ pores in the membrane.

## Materials and Methods

### Primary Cultures of Rat Hippocampal Neurons

Hippocampal neurons were obtained from 18-day embryos from pregnant Sprague-Dawley rats and cultured for 10–14 DIV as previously described (Aguayo and Pancetti, 1994).

### HEK-293 Cells Culture

HEK cells were cultivated in Dulbecco’s modified Eagle’s medium (D-MEM; Life Technologies, Carlsbad, CA, United States) supplemented with 10% fetal bovine serum (Life Technologies) and streptomycin/penicillin (200 units each, Life Technologies). Cells were maintained with 5% CO_2_ at 37°C.

### Preparation of Beta Amyloid Peptide

Human Aβ42 fluorescently labeled with FAM at the N-terminal or without fluorescence were bought from Biomatic (United States) and GenicBio (China), respectively. Oligomeric species of Aβ42 (Aβ_o_) were prepared as previously described (Peters et al., 2013). Briefly, Aβ was dissolved in HFIP (10 mg/mL) (Merck Millipore, United States) and incubated in a parafilm sealed tube at 37°C for 2 h. Then, the solution was incubated at 4°C for 20 min and aliquots of 5 μL were placed in 1.5 mL Eppendorf tubes. The tubes were left with the lids open inside the chamber to evaporate the solvent (about 20 min) and until the appearance of a thin clear film in the bottom of the tube. After evaporation was complete, aliquots were stored at -80°C. In order to dissolve the films to form oligomer-rich solution to be used in the experiments, nanopure water was added to obtain a final concentration of 80 μM and the tubes were incubated at room temperature for 20 min. Subsequently, a Teflon-coated magnetic stir bar was added to the solution (size: 2 mm × 5 mm) and stirred at room temperature (typically 22°C) at 500 rpm for 24–48 h. This solution was used to perform the experiments. To characterize the presence of Aβo in the preparations used in all the experiments, we used transmission electron microscopy coupled to immunogold staining that showed the presence of spherical or disk-shaped structures of Aβ ranging in sizes from 5 to 25 nm approximately (**Supplementary Figure S1**).

### Immunogold and Transmission Electronic Microscopy

Five microliters of Aβo, at a concentration of 50 μM, were applied to carbon-coated Formvar grids (Origen). Non-specific immunoreactivity was blocked with 3% bovine serum albumin (BSA) for 30 min at room temperature and incubated with the primary antibody anti-Aβ (1:50; Santa Cruz Biotechnology) for 1 h. A secondary 5-nm gold-conjugated anti-mouse IgG antibody was used at a 1:20 dilution for 30 min. Samples were fixed with a 2% glutaraldehyde solution for 5 min. Aβo were stained with 5 μL of 0.2 % (wt/vol) phosphotungstic acid and the grid was air-dried. Samples were examined using a JEOL 1200 EX II electronic microscope.

### Changes in Cholesterol Levels in the Cell Membrane

To increase the levels of cholesterol in the neuron and in the plasma membrane, cells were incubated with media containing MβCD/cholesterol complex (Cholesterol-Water Soluble, Sigma, United States) (Fjaervik and Zotchev, 2005). Unless otherwise stated, the concentration of cholesterol used in the experiments was 200 μM. The cells were immediately incubated in this solution for 20 min at 37°C in culture medium and washed with PBS before adding Aβ. To decrease the content of cholesterol, cells were incubated with MβCD (Sigma, United States) (Taube et al., 2009). Unless otherwise stated, the concentration used in the experiments was 3 mM. Cells were incubated for 30 min at 37°C with this solution in culture medium. Subsequently, the cells were washed with PBS and Aβ treatments were initiated. It is important to consider that both MβCD and MβCD/Cholesterol complexes likely modify the cholesterol content in internal membranes. Therefore, to quantify the changes in the membrane, we used Fillipin III (see section “Quantification of Cholesterol”) that is believed to interact mainly with membrane sterols (Aparicio et al., 2004; Karnell et al., 2005; Ng et al., 2005; Maxfield and Wustner, 2012; Payero et al., 2015). In addition, with ANEP GP imaging (see section “Di-4-ANEPPDHQ (ANEP) Staining and GP Imaging”), we only considered pixels in the peripheral plasma membrane during the analysis (Jaureguiberry et al., 2014).

### Quantification of Cholesterol

Filipin III from *Streptomyces filipinensis* (Sigma, United States), which binds with high affinity to cholesterol, was used for fluorescent quantification (Karnell et al., 2005; Jaureguiberry et al., 2014). 5 mg of Filipin III was dissolved in 400 μL of DMSO giving a stock solution of 12.5 mg/mL which was stored at -80°C. Cells were incubated for 45 min with Filipin III (50 μg/mL) at RT in PBS after post-fixation (15 min with 4% paraformaldehyde). Cells were washed with PBS and the fluorescence of each well, indicative of the level of cholesterol on the cell surface, was read on a NiovoStar plate reader (BMG Labtech, Germany) with a filter Ex = 340 nm/Em = 450 nm.

### Cell Viability Assay

After increasing or decreasing membrane cholesterol levels and subsequent Aβ treatment, cells were incubated with a MTT solution to measure cell viability. MTT was dissolved in DPBS (Gibco, United States) to 5 mg/mL and 100 μL MTT solution was added to each well to reach a final concentration of 0.45 mg/mL. Incubation was done for 2 h at 37°C. After this, a solubilization solution was added to each well to dissolve formazan crystals [solubilization solution: 40% (vol/vol) dimethylformamide in 2% (vol/vol) glacial acetic acid + 16% (wt/vol) of sodium dodecyl sulfate, pH = 4.7]. Cells were read on a NovoStar microplate photometer (BMG Labtech, Germany) at an Abs of 570 nm.

### Electrophysiology

Electrophysiological recordings were carried out using the patch clamp technique as previously described (Sepúlveda et al., 2014). Briefly, Aβ aggregates were used at 0.5–1 μM/L. Perforated recordings were obtained as follows: the perforating agent was added into the pipette solution and a 5 mV pulse was used to monitor the formation of the perforation at a holding potential of -60 mV using an Axopatch 200B amplifier (Molecular Devices, United States). Data were displayed and stored using a 1322A Digidata acquisition board and analyzed with electrophysiological pClamp 10.1 software (Molecular Devices, United States). The external solution contained the following (in mmol/L): 150 NaCl, 5.4 KCl, 2.0 CaCl_2_, 1.0 MgCl_2_, 10 glucose, and 10 HEPES (pH 7.4, 330 mOsm). The standard internal solution in the patch pipette contained the following (in mmol/L): 120 KCl, 4.0 MgCl_2_, 10 BAPTA, and 2.0 Na2-ATP (pH 7.4, 310 mOsmol). Some experiments involved an internal solution containing the NA7 peptide (20 μmol/L) (Peptide 2.0, United States).

### Immunocytochemistry

Hippocampal neurons were fixed for 15 min with 4% paraformaldehyde. Thereafter, cells were incubated with permeabilization and blocking solution with 0.1% Triton X-100 in HS:PBS 1:10 for 20 min. Monoclonal mouse anti-MAP2 (1:200; Santa Cruz Biotechnology, Dallas, TX, United States) antibody was incubated overnight, followed by incubation with a secondary anti-rabbit IgG conjugated with Cy3 (1:500; Jackson Immuno Research Laboratories, West Grove, PA, United States) for 2 h. All antibodies were diluted with horse serum (10%) in PBS. Samples were mounted in DAKO mounting medium (Dakocytomation, United States) and observed under a spectral confocal laser scanning microscope (LSM780, Zeiss, Germany) using a 63× 1.4 numerical aperture oil immersion objective (Zeiss, Germany) under the following conditions: for excitation we used two laser lines (488 nm, 561 nm) and emission was collected in the 490–540 nm and 569–610 nm ranges, respectively. 16-bit images were collected using a pixel time of 1.58 μs and a pixel size of 110 nm.

### Quantification of Number and Size of Aβ-FAM Clusters

Due to the diffraction limit of light, oligomeric species of Aβ cannot be observed by conventional laser confocal light microscope. Additionally, light undergoes diffraction while traveling in an imaging system leading to image blurring and limiting visual access to details. The blurring is characterized by a Point-Spread Function (PSF) that along with the original image can be used in a deconvolution algorithm to restore microscopic details. Therefore, using the Richardson-Lucy algorithm provided by DeconvolutionLab2 plugin (Ng et al., 2005) in ImageJ (NIH) and a theoretical PSF (based on imaging parameters), we deconvolved and analyzed the confocal micrographs of neurons treated with an oligomeric preparation of N-terminus fluorescently labeled Aβ. Deconvolution was followed by maximum intensity z-projection and background adjustment. Using the MAP-2 signal, we generated a mask to only obtain the signal of fluorescent Aβ on the neuron, where the analysis was carried out. The micrographs were used to quantify the size and number of fluorescent punctas of Aβ (or clusters) on the first 20 μm of neuronal primary processes using measuring tools from ImageJ software. We defined Aβ clustering as the process in which Aβ oligomers continue to aggregate on the surface of the membrane rendering visible structures that can be quantified by their number and size with a light microscope. At least 40 processes per condition were counted.

### Di-4-ANEPPDHQ (ANEP) Staining and GP Imaging

After the treatments to increase or decrease cholesterol content, the cells were incubated with Di-4-ANEPPDHQ (Thermo Fisher Scientific, United States). The dye was dissolved in DMSO and kept at -20°C as a stock solution. On the day of the experiment, 1 μL of stock was added to 1 mL of PBS to reach a final concentration of 1.6 μM and the cells were incubated with this solution for 45 min at 37°C. After this, the cells were rinsed to remove the excess dye, fixed for 15 min with 4% paraformaldehyde and washed with PBS. Cell imaging was carried out using spectral imaging laser scanning confocal microscopy. 16-bit images were collected using a pixel time of 3.15 μs and a pixel size of 83 nm. An excitation wavelength of 488 nm was used and two emission images were taken simultaneously in the ranges of 449–580 nm (channel 1) and 619–680 nm (channel 2). Both channels were acquired using the same imaging conditions. For image analysis, images from channels 1 and 2 were used to calculate the GP (Sánchez et al., 2007; Navarro-Lérida et al., 2015) in each pixel, obtaining the GP image (**Supplementary Figure S2**) according to the following formula and using ImageJ software (NIH):

(1) $$GP=\frac{Ich1-Ich2}{Ich1+Ich2}\,$$

Where *Ich1* and *Ich2* are the fluorescent intensity for channel 1 and channel 2, respectively. Next, Ch1 was used to create a membrane mask (**Supplementary Figure S2**). This mask was composed of the first five pixels from outside of the cell to the inside (Jaureguiberry et al., 2014) and was used to obtain the GP value corresponding to the membrane from the GP image. The histogram derived from the GP analysis provides the “Membrane Average GP value” (center of the distribution) and it can be obtained using coverage analysis (Jaureguiberry et al., 2014). For the coverage analysis, the normal distribution of the histogram was fitted to 2 Gaussian peak functions with the “Fit Multi-peaks” tool of Origin Pro 8 (Microcal, Origin Lab, Northampton, MA, United States). The average value of each new Gaussian was named GP1 and GP2, respectively (**Supplementary Figure S2**), and the percentage of coverage of each Gaussian with respect to the original distribution was calculated (expressed as “Area of GP”) (**Supplementary Figure S2**).

### Data Analysis

All data obtained from the measurements of capacitive current, fluorescence and all other parameters were analyzed and plotted using OriginPro 8.0 (Microcal, Origin Lab, Northampton, MA, United States). Experiments with MβCD and the MβCD/Cholesterol complex showed that they did not have any effect on their own, so unless otherwise indicated, the control condition reported in the study was considered in the presence of the modulator. Membrane charge was calculated by integrating the transient capacitive current after subtracting the pipette capacitance. Under this condition, the area under the capacitive current represented the membrane charge transferred (fC). Then, we fitted the current with a standard exponential function using Chebyshev algorithm to calculate the tau for the decay of the current. Using this strategy, we calculated the total charge transferred to the membrane under the patch pipette. The charge transferred curves were fitted using a dose–response function algorithm. Unless otherwise indicated, the results, including image analysis, are presented as mean ± SEM from at least five to eight neurons or cells. Statistical differences were determined using 1-way ANOVA or paired Student’s *t*-tests, followed by the Bonferroni *post hoc* test in some cases. A probability level (*p*) less than 0.05 was considered statistically significant.

## Results

### Modification of the Relative Levels of Cholesterol in Hippocampal Neurons and HEK-293 Cells and Evaluation of Aβ Toxicity

**FIGURE 1:**
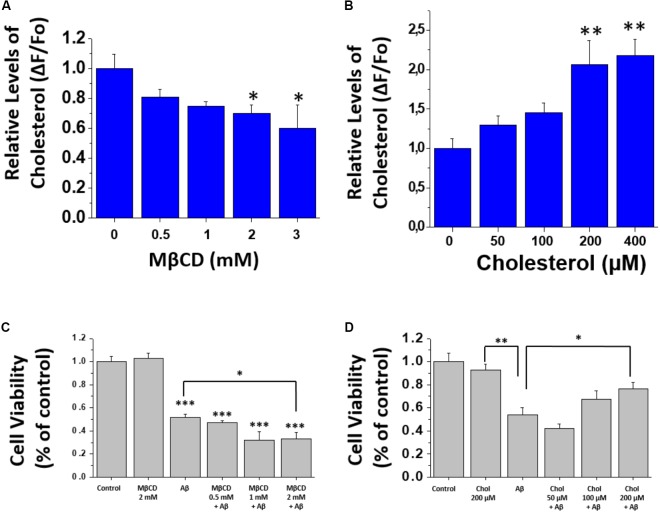
Modification of cholesterol levels in hippocampal neurons. **(A,B)** Quantification of filipin III fluorescence showing relative levels of cholesterol after treatment with MβCD (0.5–3 mM), and after treatment with different concentrations of soluble cholesterol (0–400 μM) in hippocampal neurons. **(C,D)** Cell viability assay performed after treatments to modify membrane-cholesterol and subsequent Aβ incubation (1 μM for 24 h). The bars represent the mean ± SEM. Asterisks denote: ^∗^*p* < 0.05, ^∗∗^*p* < 0.005, ^∗∗∗^*p* < 0.001.

In order to decrease the amount of cholesterol in the membranes, cells (HEK-293 cells and hippocampal neurons) were incubated with different concentrations of MβCD for 30 min, washed and then incubated with Filipin III for 45 min. The relative levels of cholesterol in the membranes were quantified by measuring the fluorescence of Filipin III in the well with the cells under treatment. The data show that the treatment produced similar effects in both hippocampal neurons (**Figure 1A**) and HEK-293 cells (**Supplementary Figure S3A**). Also, a significant decrease in the total fluorescence in the wells was observed in both cell types, indicating that 3 mM of MβCD was capable of removing cholesterol from the membrane. In another series of experiments, we increased the level of cholesterol by using soluble cholesterol that has been shown to incorporate easily into cell membranes. Cells were incubated for 20 min with a solution that contained soluble cholesterol (MβCD/cholesterol complex), and afterward, incubated with Filipin III for 45 min. The results show an increase in the relative levels of cholesterol in the membrane of hippocampal neurons (**Figure 1B**) and HEK-293 cells (**Supplementary Figure S3B**). Taken together, these results demonstrate that the treatments were capable of effectively decreasing or increasing the levels of cholesterol in cell membranes.

Next, we wanted to determine if changes in the level of cholesterol affected cell viability by itself and after the addition of Aβ. For this, we examined the viability of hippocampal neurons under the same experimental conditions used previously to increase or decrease cholesterol levels followed by a more prolonged treatment with Aβ (1 μM) for 24 h. Subsequent evaluation was done using an MTT assay. The results in **Figure 1C** show that MβCD-treatment (2 mM) did not produce any changes in cell viability. Notably, when neurons previously treated with MβCD (2 mM) were incubated with Aβ, cell viability was reduced to 32 ± 5%, while treatment with Aβ alone reached 52 ± 2% of the control condition (**Figure 1C**). On the other hand, after culturing the neurons with cholesterol, we found an increase in cell viability in a concentration-dependent manner (50–200 μM), even in the presence of Aβ. In the absence of added cholesterol, Aβ decreased neuronal viability in 47 ± 5% and it increased to 75 ± 4% in the presence of cholesterol (**Figure 1D**). These data show that reducing cholesterol renders the neurons more sensitive to Aβ-induced toxicity. On the other hand, cholesterol addition produced a protective effect on Aβ toxicity (**Figure 1D**).

### Decreased Cholesterol Content Diminished Association of Aβ Aggregates in Hippocampal Neurons

**FIGURE 2:**
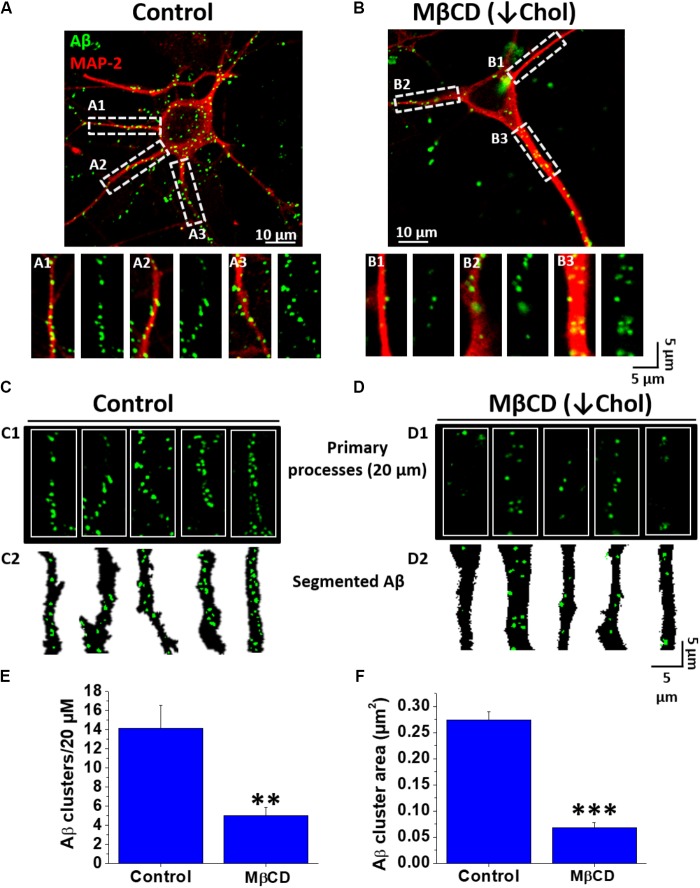
Decreased Aβ association to neurons after lowering cholesterol. **(A,B)** Confocal microscopy images of hippocampal neurons exhibiting fluorescence for MAP-2 (red) and demonstrating the distribution of Aβ_42_-FAM (green) aggregates on the cell membrane in control and after treatment with MβCD (3 mM) for 30 min. **(A1–A3,B1–B3)** ROIs of representative neurites showing the overall distribution of Aβ (1 μM, after 1 h treatment) on neuronal processes. **(C,D)** Representative traces of primary processes shown in **(A,B)**, respectively. **(E,F)** Quantification of Aβ clusters (number and area) in the first 20 μm of hippocampal neuron primary processes showing that both parameters decreased after treatment with MβCD (3 mM) for 30 min. The bars represent the mean ± SEM. Asterisks denote: ^∗∗^*p* < 0.005 and ^∗∗∗^*p* < 0.001.

Next, we decided to evaluate if the cholesterol present in neuronal membranes was able to modulate the association of Aβ to the cells. To do this, hippocampal neuron cultures were pre-treated with and without MβCD to decrease the content of cholesterol in cellular membranes and then incubated for 1 h with fluorescently labeled Aβ (Aβ-FAM). The results in **Figure 2** show that decreasing the levels of cholesterol in the cell membrane diminished the membrane association of Aβ (green signal). To obtain quantitative data from these images, we defined the term “Aβ cluster” as the Aβ signal obtained in confocal images (see sections “Materials and Methods” and “Quantification of Number and Size of Aβ-FAM Clusters”), and we quantified the area and number of these clusters in the first 20 μm of primary processes of the neurons in culture. In control conditions, confocal images showed the association of the fluorescent peptide in a punctuate pattern on the neuron to the soma and particularly on the neurites (**Figure 2A**, green signal). The same pattern was observed in the cholesterol-depleted neurons, however, the level of peptide that associated with the cells was significantly reduced (**Figure 2B**). Examining a zoomed ROI (region of interest) in the original image, we were able to examine in more detail the association of Aβ with the primary processes of hippocampal neurons in both conditions (**Figures 2C1,D1**). The segmented signals of Aβ obtained in these ROIs are shown in order to better see and understand the concept of cluster and what we quantified in both conditions (**Figures 2C2,D2**). Primary processes of neurons treated with MβCD exhibited a marked decrease in the association of Aβ clusters (**Figures 1C,D**). Quantification of the fluorescent Aβ signal revealed that the clusters were diminished in number by ∼ 65% (Control: 14.1 ± 2.3 vs. MβCD: 5.0 ± 0.8) (**Figure 2E**). The size (area) of these clusters was also decreased by ∼ 75% from 0.27 ± 0.01 μm^2^ in the control condition to 0.06 ± 0.01 μm^2^ in the depleted-cholesterol condition (**Figure 2F**). These results indicate that a decreased content of cholesterol in the membrane prevented Aβ association with neurons; diminishing the size and number of membrane-associated Aβ clusters.

### Increased Membrane Cholesterol Levels Augmented Association of Aβ Aggregates in Hippocampal Neurons

**FIGURE 3:**
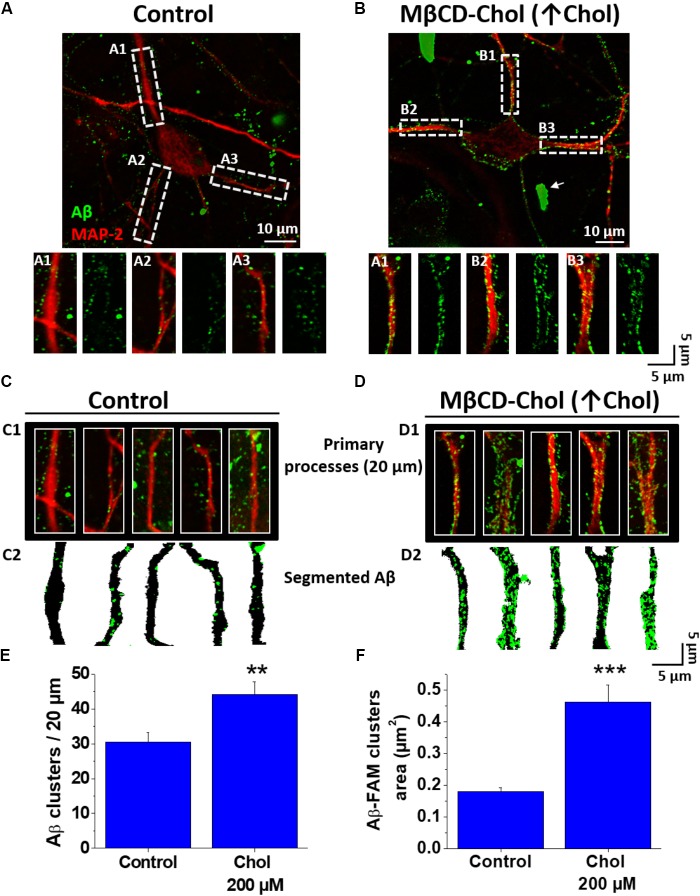
Increased cholesterol promotes association of Aβ to neurons. **(A,B)** Confocal microscopy images of hippocampal neurons exhibiting fluorescence for MAP-2 and demonstrating the distribution of Aβ_42_-FAM aggregates (green) on the cell membrane after treatment with MβCD-Cholesterol complex (200 μM) for 20 min. **(A1–A3,B1–B3)** ROIs of representative neurites show the overall distribution of Aβ (1 μM, after 1 h) on neuronal processes in more detail. **(C,D)** Representative traces of primary processes shown in **(A,B)**, respectively. **(E,F)** Quantification of Aβ clusters (number and area) in the first 20 μm of hippocampal neuron primary processes showing that both parameters were increased after treatment with MβCD-Cholesterol complex (200 μM) for 20 min. The bars represent the mean ± SEM. Asterisks denote: ^∗∗^*p* < 0.005 and ^∗∗∗^*p* < 0.001.

To obtain a better understanding of cholesterol influence on the Aβ membrane association, we performed the opposite experiment (i.e., increased the levels of cholesterol using a MβCD-cholesterol complex as a donor of cholesterol). The neurons treated with cholesterol showed a significant increase in Aβ association compared with the control condition (**Figures 3A,B**). Interestingly, we also found some large aggregates associated with the neuronal tissue (**Figure 3B**, white arrows). The analysis of the segmented data showed that the cluster number and size increased in the presence of supplemental cholesterol (**Figures 3C1–D2**). For instance, in cholesterol supplemented neurons, we found an increase of ∼45% in the number of Aβ clusters compared to those obtained in control conditions (clusters number/20 μm, control: 30.5 ± 2.6 vs. MβCD-cholesterol (↑ cholesterol): 44.1 ± 3.6; **Figure 3E**). Interestingly, the difference was particularly more significant when we quantified the area of these clusters [Aβ cluster area (μm^2^)]. For example, the data revealed that cluster area in control conditions was 0.18 ± 0.01 vs. an average value of 0.46 ± 0.05 in MβCD-cholesterol treatment (↑ cholesterol), representing an approximately 150% increase in cluster area due to the treatment with cholesterol as compared to control (**Figure 3F**).

### Increase in Cholesterol Reduced the Membrane Perforation Induced by Aβ

**FIGURE 4:**
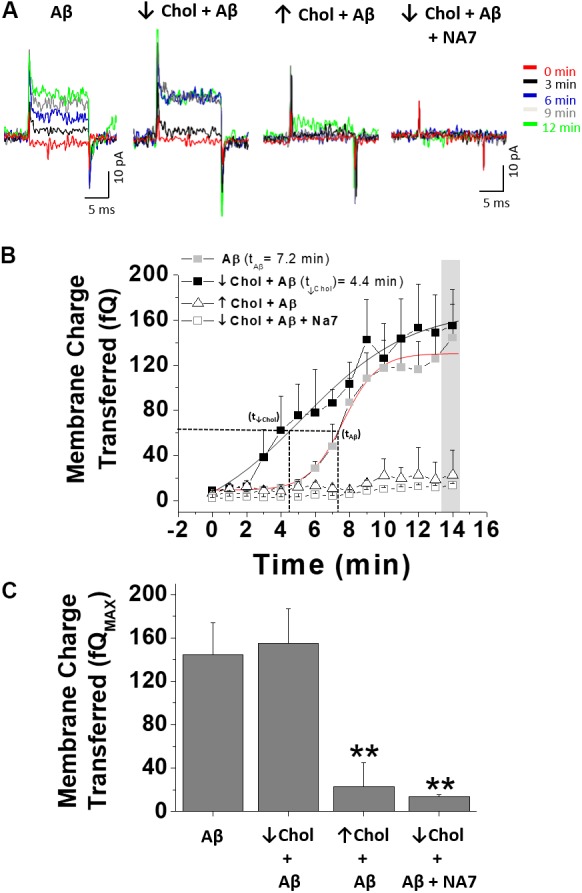
The effect of Aβ on membrane integrity depends on the level of cholesterol. **(A)** Representative traces of capacitive currents recorded at different times after increasing or decreasing relative levels of cholesterol in HEK-293 cells. **(B)** Time course of Aβ-induced perforation for each of the conditions described in **(A)** showing the kinetic of the perforation process and how this effect changes when membrane-cholesterol levels are modified. **(C)** Quantification of membrane charge transferred at the end of the registration period. The effects of Aβ in a cholesterol-depleted condition were blocked by the NA7 peptide. Bars represent the mean ± SEM. Asterisk denotes ^∗∗^*p* < 0.005.

Based on the above findings, we wanted to examine how increasing or decreasing cholesterol in the cell membrane affected the toxic action of Aβ (perforation) in HEK-293 cells, similar to the effects in hippocampal neurons (Sepúlveda et al., 2014). To examine this membrane phenomenon, we used the patch-perforated technique and applied Aβ through the glass electrode. Under this experimental condition, Aβ comes in contact with the membrane and if it is capable of perforating it, a change in the capacitive current occurs. In **Figure 4A**, a typical recording of a cell patched with Aβ exhibiting the capacitive current is shown (**Figure 4A**, first trace, orange). The amplitude of this current increased with time of exposure, reaching a maximal after 10 min of recording (**Figure 4A**, first recording, green trace). The data also shows the effect of decreasing or increasing cholesterol on the changes in the amplitude of the capacitive currents (**Figure 4A**, second and third panels). The data shows that in presence of supplemental cholesterol, the effects of Aβ on membrane perforation were significantly reduced. The graph in **Figure 4B** illustrates the time course of the effect of Aβ in terms of charge transferred under the conditions shown in **Figure 4A**. The data show that Aβ initiated its effects at 5 min in control cells, increasing the charge transferred and reaching a maximum at 15 min (**Figure 4B**, gray filled squares). On the other hand, when the recordings were made in cholesterol-depleted cells, the effect of Aβ perforating the membrane was accelerated (**Figure 4B**, black filled squares). For example, the half-time for maximal charge transferred in cells treated with MβCD was 4.4 min (t_↓Chol_) which compares to 7.2 min (t_Aβ_) in control cells, showing that the cell membrane was perforated much faster by Aβ when cholesterol was depleted in the membrane. Interestingly, increased levels of cholesterol produced the opposite effect, blocking the perforating effect of Aβ (**Figure 4B**, white triangles with black outline). These findings suggest that the effect of Aβ depends on the amount of cholesterol in the membrane. Finally, to validate that the effect of Aβ was related to the formation of pores in the membrane, we performed experiments with NA7 (Arispe, 2004; Arispe et al., 2007), a mini-peptide that has been widely studied in our and other laboratories and is capable of interfering with the formation of amyloid pores in cell membranes. Therefore, we decided to use the condition with low cholesterol that produced facilitation in the perforation, finding that under these conditions the formation of the pore was inhibited (**Figure 4A**, fourth panel; **Figure 4B**, white squares with gray outline). At the end of the recordings (i.e., 15 min), we quantified the amount of membrane charge transferred in each of the assayed conditions. As shown in **Figure 4C**, the amount of charge transferred for the conditions with Aβ alone was very similar to the condition in which we diminished cholesterol and then applied Aβ. Also, the presence of cholesterol in the membrane almost completely inhibited the perforation by the end of the experiment. This result was similar to the condition in which the MβCD-facilitated perforation was inhibited by the NA7 (**Figure 4C** compare bar 2 with bar 4).

### Changes in Membrane Fluidity Induced by Treatments With MβCD and Cholesterol

In the previous results, we found that Aβ-mediated membrane perforation was dependent on the level of membrane cholesterol. Because cholesterol is an essential component of cell membranes and modulates membrane fluidity (Vestergaard et al., 2010), we thought that this biophysical property might be playing an important role in the membrane actions mediated by Aβ. Therefore, we decided to test if membrane fluidity was affected under the same experimental conditions previously used. In order to do this, neurons were treated with a dye, di-4-ANEPPDHQ (Owen and Gaus, 2010), after increasing or depleting cholesterol. The emission spectrum of this fluorescent dye is different if the molecule is located in areas where the lipids are ordered or disordered, presenting a blue shift when lipids are organized (water content is reduced). The generalized polarization (GP) function is the mathematical quantification of this spectral shift (see sections “Materials and Methods” and “Di-4-ANEPPDHQ (ANEP) Staining and GP Imaging”): high GP values are related with ordered lipids and vice versa.

The level of hydration in the membrane is related with membrane fluidity (Parasassi et al., 1997). Therefore, we refer to membrane fluidity in terms of “membrane water content” and we express this as a GP value (see sections “Materials and Methods” and “Di-4-ANEPPDHQ (ANEP) Staining and GP Imaging”).

**FIGURE 5:**
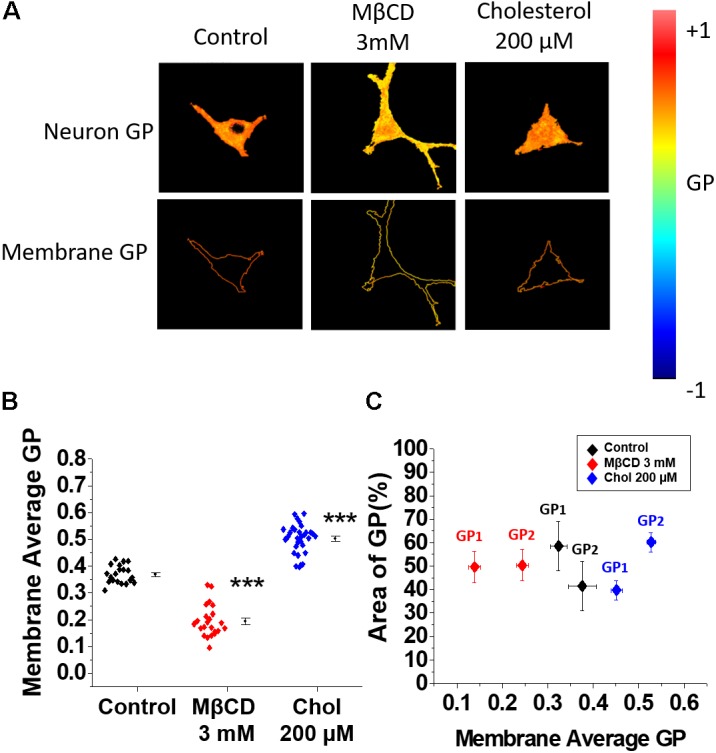
Membrane fluidity changes after decreasing or increasing cholesterol levels. **(A)** Representative micrographs of hippocampal neurons showing the distribution of scaled GP values in the entire cell and in the membrane area under the experimental conditions previously described. **(B)** Average GP values showing a decrease in GP in membranes when neurons were treated with MβCD 3 mM. The opposite effect was found when the cells were incubated with 200 μM of soluble cholesterol for 20 min. **(C)** Coverage analysis showing GP changes in two populations of the membrane domains (GP1 and GP2) for each of the conditions described in **(B)** In the presence of 3 mM MβCD both domains exhibit lower GP values (more fluid) compared to control conditions, while the addition of cholesterol produce dehydration of both lipid domain populations, becoming more rigid and exhibiting higher GP values. The graphs represent the mean ± SEM. Asterisks denote: ^∗∗∗^*p* < 0.001.

The results in **Figure 5A** show the GP images from the whole neurons obtained under control and treated conditions (MβCD and cholesterol). Qualitative analysis of the images showed changes in fluidity after the two treatments: an increase (yellow color) after MβCD and a decrease (orange color) after cholesterol treatment. The quantitative analysis using GP values (**Figure 5B**) showed a significant decrease in the average GP values in MβCD-treated cells (0.19 ± 0.01) with respect to control cells (0.36 ± 0.01) (**Figure 5B**). We interpreted this result as a decrease in the order of the membrane since cholesterol was removed, augmenting membrane fluidity. On the other hand, the GP value increased ∼40% when the levels of membrane cholesterol were incremented (0.50 ± 0.01), interpreted as cholesterol dehydrating the membrane, decreasing membrane fluidity (**Figure 5B**).

Next, a coverage analysis was made to evaluate the relationship between the average changes in fluidity with the possible changes in lipid domains in the membrane. The coverage analysis describes GP changes in terms of two populations of membrane domains having a high average GP (less fluid) and low average GP value (more fluid). **Figure 5C** shows that the average GP value of the control cells (0.36 ± 0.01) is the result of two populations that exhibited GP values of 0.32 ± 0.02 (GP1) and 0.38 ± 0.03 (GP2) with 58.5 and 41.4% area coverage, respectively (**Figure 5C**, black rhombuses). The observed decrease in average GP value after incubation with MβCD was due to changes in the distribution and fluidity of the two populations of lipid domains: both populations became more fluid when cholesterol was removed (GP1 = 0.13 ± 0.01 and GP2 = 0.24 ± 0.01) and the percentage of coverage for both GP1 and GP2 changed to 50% (**Figure 5C**, red rhombuses). Cholesterol addition produced a dehydration of both populations of lipid domains reversing the percentage area coverage as compared to control conditions: GP1 = 0.45 ± 0.01 and GP2 = 0.52 ± 0.01 with a percentage of coverage for GP1 and GP2 of 39.7 and 60.2%, respectively (**Figure 5C**, blue rhombuses). These results suggest that both domains (GP1 and GP2) became less fluid, which is expected since it has been reported that when cholesterol is inserted in the bilayer it produces dehydration making the membrane more rigid (Ayee and Levitan, 2016).

## Discussion

The role of Aβ in the neurotoxicity leading to AD has been debated since its discovery. For example, previous studies have indicated that membrane surface components can interact with extracellular Aβ (Sasahara et al., 2013; Qiang et al., 2014; Jamasbi et al., 2018). As a result of this interaction, Aβ would be inserted into the membrane forming multimeric structures able to perforate the membrane and disrupt the ionic homeostasis of the cell (Sepúlveda et al., 2014; Fernández-Pérez et al., 2017). However, disease progression does not seem to be highly dependent on amyloid Aβ, and several other neurotoxic factors appear to interact with each other. For instance, the activation of glutamatergic neurotransmission with kainate resulted in an increased cholesterol concentration in the rat hippocampus (Ong et al., 2010). On the other hand, another study on hippocampal neurons found that at 30 min, the time frame of our experiments, stimulation of glutamatergic neurotransmission induced a loss of membrane cholesterol and altered calcium signaling (Sodero et al., 2012). The reduction in membrane cholesterol was related to an associated intracellular calcium dyshomeostasis that was prevented by the addiction of exogenous cholesterol. Therefore, the initiation of the disease appears to be linked to the amyloid cascade, but its progression is potentiated by a number of other cellular and molecular factors making it difficult to find an efficient treatment.

In the present study, we found that cholesterol has a protective effect in the initial stages of Aβ toxicity. For example, increasing the level of cholesterol in the membrane augmented the number and size of Aβ clusters associated with the membrane, preventing neurotoxicity. This effect could be associated with changes in the hydrophobic environment of the membrane itself due to the presence of more cholesterol favoring Aβ clustering, and therefore, the size of the observed microscopic clusters. It is known that hydrophobicity plays a role in the assembly of Aβ (Linse et al., 2007; Fernandez-Perez et al., 2016) because it can affect the conformation of the peptide itself, allowing it to form larger aggregates (Giacomelli and Norde, 2003). Indeed, the membrane environment favored by the presence of cholesterol appears to be crucial for Aβ aggregation (Yu and Zheng, 2012).

**FIGURE 6:**
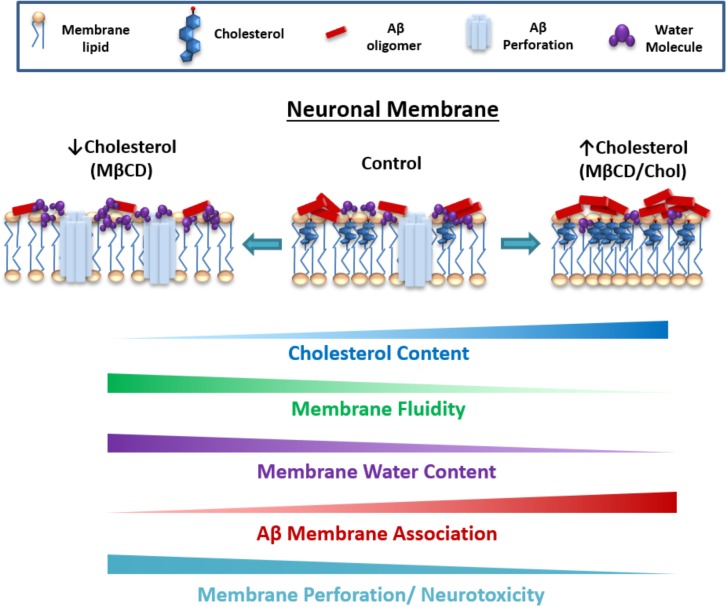
Proposed model for the role of cholesterol content and membrane fluidity in the early neurotoxic actions of Aβ. Cholesterol augmentation reduces fluidity and water content of the membrane and favors Aβ association (clusters) on the surface of the bilayer. In parallel, cholesterol appears to attenuate Aβ perforation, possibly making it difficult for the peptide to insert in the membrane because of the presence of more rigid domains. On the contrary, increase in membrane fluidity with reduced cholesterol appears to reduce Aβ association, making the membrane more susceptible to be disrupted by smaller Aβ conformations (oligomers).

It could be expected that an increase in Aβ in the membrane would favor the process of perforation, but this was not observed. Actually, there was a negative correlation between the burden of Aβ on the cell and the membrane perforation. We found that membrane perforation was significantly attenuated when the neurons were enriched with cholesterol. After changing the level of cholesterol in the neurons, we found that the membrane itself underwent physicochemical changes that might be impeding the insertion of the peptide and the perforation. Indeed, our data showed a decrease in membrane fluidity with high cholesterol (i.e., more packed and more rigid) that could explain why the peptide might not be able to perforate the cell bilayer, diminishing neurotoxicity (**Figure 6**). This is in agreement with previous results indicating that because cholesterol increases the rigidity of the membrane, it inhibits the formation of pores (Allende et al., 2005). Moreover, cholesterol also blocked the increase in intracellular calcium concentrations produced by Aβ, preventing its cytotoxic effects (Zhou and Richardson, 1996; Mizuno et al., 1999; Kawahara and Kuroda, 2001). Hence, cholesterol might be a neuroprotective factor against the toxicity of Aβ oligomers in the initial stages of the disease, but may account for the later on-set of this illness (Cecchi et al., 2009). This is consistent with previous studies showing that the disruption in cholesterol homeostasis could be of vital importance to the cell since toxic Aβ aggregates interact more easily with membranes having lower cholesterol (Kawahara et al., 2000; Yip et al., 2001).

Furthermore, the decrease in membrane fluidity could also be a component that favors the increase in the size of the observed Aβ particles (i.e., Aβ clusters). For example, when the membrane is more packed it might prevent perforation by impeding the insertion of Aβ in the membrane and favoring its accumulation on the surface (**Figure 6**). It is interesting to speculate that as there is more peptide present on the surface of the bilayer, its oligomerization is favored and more and larger aggregates can form on the membrane surface, which along with the high content of cholesterol, serves as a hydrophobic core to continue the seeding and its accumulation (Mizuno et al., 1999; Kakio et al., 2001) (**Figure 6**). Alternatively, it is possible that larger clusters are less neurotoxic than the ones present when cholesterol levels are low. It is also possible that the high Aβ burden detected in asymptomatic individuals might be associated with high levels of brain cholesterol, acting as a neuroprotector (Mintun et al., 2006; Rowe et al., 2007).

There are several studies that resulted in controversial results on the impact of Aβ accumulation under different stages of membrane cholesterol (discussed below). For example, we found that using β-cyclodextrin to remove membrane cholesterol decreased Aβ association, and although this may seem to be the perfect condition to avoid Aβ toxicity, our results actually show that the absence of cholesterol favors Aβ membrane disruption. This is because we believe that a decreased amount of cholesterol is favoring the insertion of the peptide, allowing it to become more toxic (**Figure 6**). Interestingly, this finding is in line with a previous study that showed that the hippocampus of AD subjects displayed a significant reduction in membrane cholesterol (Ledesma et al., 2003). However, this point has been controversial, since there is evidence that shows the contrary. For example, the reduction in cholesterol by subcutaneous injection of β-cyclodextrin in Tg19959 mice for 4 months showed a significant decrease in Aβ, as well as improved memory and a decrease in phosphorylated tau pathology (Yao et al., 2012). This may account for the cytotoxicity observed in other studies when membrane cholesterol was incremented (Parasassi et al., 1997; Ayee and Levitan, 2016; Jamasbi et al., 2018). Another study also described that cholesterol was a promoter of Aβ toxicity (Lin et al., 2008), and it has been shown that an increased amount of cholesterol in the brain aggravates the neurodegenerative process in an AD-like pathology (Djelti et al., 2015). Additionally, an APP/PS1 mouse model overexpressing the regulatory element-binding protein-2 (SREBP-2) truncated mutation, which exhibits high cholesterol levels in the brain, also exhibits more synaptic degeneration and neuronal death compared to control mice with only APP/PS1 mutations (Barbero-Camps et al., 2013). Some studies have intended to explain this by suggesting that the oxidation of cholesterol is responsible for the toxicity of Aβ when the levels of this lipid are increased in the membrane (Peters et al., 2013; Jaureguiberry et al., 2014).

Based on some of the previous discussed results that described that high cholesterol levels may promote Aβ toxicity, several strategies have been developed to reduce brain cholesterol levels through the use of statins. Husain et al. (2017) showed that rosuvastatin inhibited Aβ aggregation and ameliorated cognitive impairment in an *in vivo* study. Additionally, although lovastatin was able to diminish Aβ levels *in vitro*, it was unable to produce any effect on Aβ levels in the brains of the mice, even though the mevalonate pathway was significantly modified (Mendoza-Oliva et al., 2015). Conversely, Tg2576 mice that were treated with lovastatin (the most brain permeable statin) showed an increase in Aβ production and senile plaque deposition (Park et al., 2003). Significantly, several trials have been performed in AD patients to examine if cholesterol reduction can contribute to a diminished progression of the disease (Miida et al., 2004; Wanamaker et al., 2015). Unfortunately, the most recent large-scale, randomized double-blind placebo-controlled clinical trials with atorvastatin (Feldman et al., 2010) and simvastatin (Mintun et al., 2006) failed to slow disease progression in mild-to-moderate AD. Nevertheless, it is important to keep in mind that in the symptomatic phase the brain pathology appears largely irreversible, rendering any treatment non-viable on patients with advanced brain disease.

Another matter for current discussion is the role that dietary cholesterol might play in neurodegeneration. This has been somewhat demonstrated previously with the use of mice fed with a high cholesterol diet that suggested that hypercholesterolemia accelerated the cognitive deficits induced by Aβ (Refolo et al., 2000; Park et al., 2013; Umeda et al., 2012). However, it is still unresolved if this sort of cholesterol action is due to a vascular or a direct action on the brain. For instance, it was shown that a cholesterol-enriched diet could compromise the blood–brain barrier (BBB) (Yip et al., 2001) leading to brain parenchyma damage (Ullrich et al., 2010). Perhaps the presence of lipoproteins with unesterified cholesterol could be another factor in Aβ neurotoxicity (Ghribi et al., 2006; Dias et al., 2014). Indeed, although these mice exhibit high cholesterol brain levels, they also exhibit a clear vascular pathology. Hence, Aβ might be causing an enhanced neuronal death because the brain is already injured.

Finally, the role of cholesterol in AD has been previously studied at different levels of complexity making it difficult to fit all the results into one single mechanism. In the present study, we looked at cellular and biophysical approaches to examine the mechanisms underlying the physiological data on neurotoxicity. Cholesterol appears to have a membrane protective action for very early toxic action on neuronal membranes suggesting a new mechanistic role of membrane fluidity during the initial stages of the disease.

## Conclusion

(1) Reduction in membrane cholesterol diminished membrane fluidity. This was accompanied by a reduction in Aβ clustering and association, but facilitated membrane disruption actions and enhanced Aβ neurotoxicity.
(2) On the other hand, increasing the levels of this membrane constituent produced a dehydration of the lipid bilayer and an attenuation of Aβ perforation, but enhanced the clustering and association to the neuronal membrane.
(3) In summary, cholesterol content and fluidity can regulate the distribution, insertion and subsequent rupture of the neuronal membrane by the Aβ peptide, suggesting that lipid composition, plasma membrane organization and fluidity are crucial bilayer properties for the neurotoxic actions of Aβ.

## Ethics Statement

Animal use protocols were approved by the University of Concepción Bioethics Committee.

## Availability Of Supporting Data

The datasets used and/or analyzed during the current study are available from the corresponding author on reasonable request.

## Author Contributions

LA, RP, SS, BV, EF-P, FS, CP, DB, NR-L, and JG-S contributed to experimental design. EF-P, FS, CP, DB, NR-L, and JG-S carried out the study and analyzed data. LA and EF-P wrote the manuscript. All authors read and approved the final manuscript.

## Conflict of Interest Statement

The authors declare that the research was conducted in the absence of any commercial or financial relationships that could be construed as a potential conflict of interest.

## Supplementary Material

FIGURE S1 Electron micrographs that demonstrate the presence of amyloid aggregates in the preparations used with immunogold (5 nm particle). **(A)** Micrographs of Aβ oligomers preparations showing the distribution of different size oligomers. **(B)** Zoom of the section shown in **A**, demonstrating the presence of spherical or disc shaped oligomeric species.

FIGURE S2 GP image analysis workflow. Representative scheme showing the different steps for the quantification of GP values (GP total, GP1, and GP2) in the cellular membrane. First, channel one (Ch1) is used to create the mask (taking the first 5 pixels from outside of the cell to the inside). After this, the mask is applied in the GP image obtaining the GP image for the cell membrane. From this image, we obtain the histogram and from that we obtain the GP total, GP1, and GP2. The percentage of coverage of GP1 and GP2 was calculated and expressed as “Area of GP”.

FIGURE S3 Effects of MβCD and water-soluble cholesterol in HEK-293 cells. **(A)** Quantification of Filipin III fluorescence indicating the relative cholesterol levels after treatment with MβCD (3 mM) and cholesterol/ MβCD complex (200 μM cholesterol) in HEK-293 cells **(B)**. The graphs show the effectiveness of the treatment to increase or decrease membrane cholesterol levels in this cell line. The bars represent the average ± SEM. ^∗^Denotes *p* < 0.05 and ^∗∗∗^*p* < 0.001.

## Abbreviations

Aβ: beta-amyloid peptide
AD: Alzheimer’s disease
ANOVA: analysis of variance
APOE: apolipoprotein E
APP: amyloid precursor protein
DIV: days *in vitro*
DRMDs: detergent resistant membrane domains
FAM: 5 - (and - 6) - carboxyfluorescein
GP: general polarization value
HFIP: 1,1,1,3,3,3-hexafluoro-2-propanol
MβCD: methyl-beta-cyclodextrin
MβCD/cholesterol: methyl-beta-cyclodextrin/cholesterol complex
MTT: (3-(4,5-dimethylthiazol-2-yl)-2,5-diphenyltetrazolium bromide)
SEM: standard error of the mean
