# Supplementary figures and images for: Effect of Cholesterol on Membrane Fluidity and Association of Aβ Oligomers and Subsequent Neuronal Damage: A Double-Edged Sword
Source: Front Aging Neurosci. 2018 Aug 3;10:226. doi: 10.3389/fnagi.2018.00226 (PMC6085471; doi:10.3389/fnagi.2018.00226)

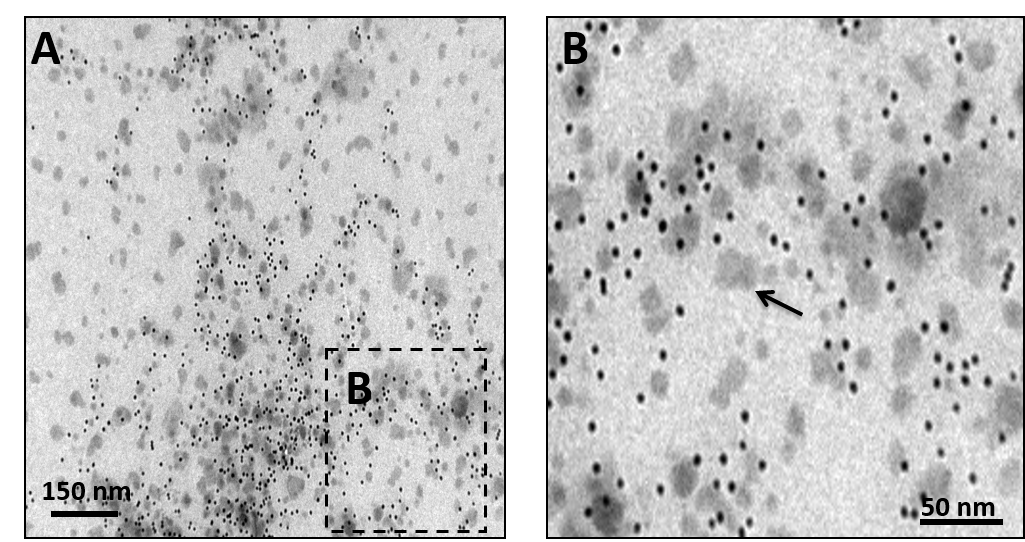

Supplement: FIGURE S1 — Electron micrographs that demonstrate the presence of amyloid aggregates in the preparations used with immunogold (5 nm particle). (A) Micrographs of Aβ oligomers preparations showing the distribution of different size oligomers. (B) Zoom of the section shown in A, demonstrating the presence of spherical or disc shaped oligomeric species. [file Image_1.TIF]

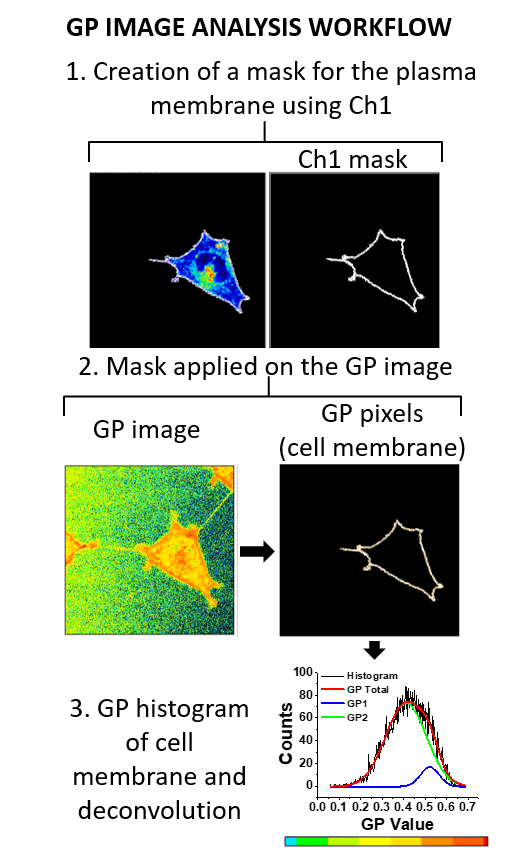

Supplement: FIGURE S2 — GP image analysis workflow. Representative scheme showing the different steps for the quantification of GP values (GP total, GP1, and GP2) in the cellular membrane. First, channel one (Ch1) is used to create the mask (taking the first 5 pixels from outside of the cell to the inside). After this, the mask is applied in the GP image obtaining the GP image for the cell membrane. From this image, we obtain the histogram and from that we obtain the GP total, GP1, and GP2. The percentage of coverage of GP1 and GP2 was calculated and expressed as “Area of GP”. [file Image_2.tif]

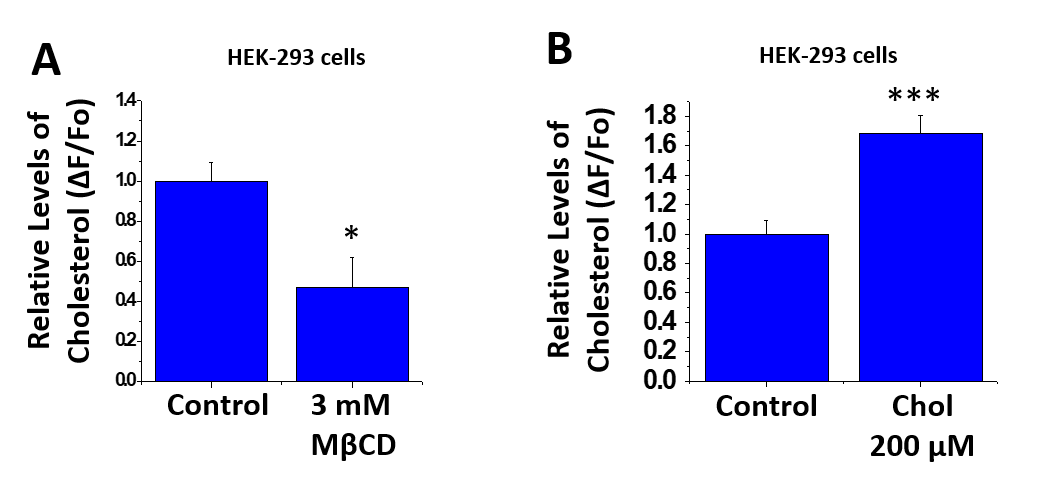

Supplement: FIGURE S3 — Effects of MβCD and water-soluble cholesterol in HEK-293 cells. (A) Quantification of Filipin III fluorescence indicating the relative cholesterol levels after treatment with MβCD (3 mM) and cholesterol/ MβCD complex (200 μM cholesterol) in HEK-293 cells (B). The graphs show the effectiveness of the treatment to increase or decrease membrane cholesterol levels in this cell line. The bars represent the average ± SEM. ∗Denotes p < 0.05 and ∗∗∗p < 0.001. [file Image_3.TIF]
